# *Satellitosis*, a Crosstalk between Neurons, Vascular Structures and Neoplastic Cells in Brain Tumours; Early Manifestation of Invasive Behaviour

**DOI:** 10.3390/cancers12123720

**Published:** 2020-12-11

**Authors:** Prospero Civita, Ortenzi Valerio, Antonio Giuseppe Naccarato, Mark Gumbleton, Geoffrey J. Pilkington

**Affiliations:** 1 Brain Tumour Research Centre, Institute of Biological and Biomedical Sciences (IBBS), School of Pharmacy and Biomedical Sciences, University of Portsmouth, Portsmouth PO1 2DT, UK; 2 School of Pharmacy and Pharmaceutical Sciences, College of Biomedical and Life Sciences, Cardiff University, Cardiff CF10 3NB, UK; gumbleton@cardiff.ac.uk; 3 Department of Translational Research and New Technologies in Medicine and Surgery, Pisa University Hospital, 56100 Pisa, Italy; valerio.ortenzi@ao-pisa.toscana.it (O.V.); giuseppe.naccarato@med.unipi.it (A.G.N.); 4 Division of Neuroscience, Department of Basic and Clinical Neuroscience, Institute of Psychiatry & Neurology, King’s College London, London SE5 9RX, UK

**Keywords:** brain tumour, satellitosis, glioblastoma, tumour heterogeneity, perineuronal satellitosis, perivascular satellitosis, invasion

## Abstract

**Simple Summary:**

This article reviews the concept of cellular *satellitosis* as originally described histologically by Santiago Ramón y Cajal in 1899 and Hans Joachim Scherer, more specifically in the context of glioblastoma invasiveness, during the early part of the 20th century. With the advent of new and emerging molecular technologies in the 21st century, the significance of both vascular and neuronal satellitosis by neoplastic cells offers intriguing possibilities into further clarifying the development, pathobiology and therapy of malignant glioma through closer investigation into the nature of these histological hallmarks.

**Abstract:**

The secondary structures of Scherer commonly known as perineuronal and perivascular satellitosis have been identified as a histopathological hallmark of diffuse, invasive, high-grade gliomas. They are recognised as perineuronal satellitosis when clusters of neoplastic glial cells surround neurons cell bodies and perivascular satellitosis when such tumour cells surround blood vessels infiltrating Virchow–Robin spaces. In this review, we provide an overview of emerging knowledge regarding how interactions between neurons and glioma cells can modulate tumour evolution and how neurons play a key role in glioma growth and progression, as well as the role of perivascular satellitosis into mechanisms of glioma cells spread. At the same time, we review the current knowledge about the role of perineuronal satellitosis and perivascular satellitosis within the tumour microenvironment (TME), in order to highlight critical knowledge gaps in research space.

## 1. Introduction

One of the first histological descriptions of *satellitosis* in the nervous system was reported by Santiago Ramón y Cajal in 1899, when he described aggregates of glial cells surrounding both the cell body of neurons and its dendrites in healthy peripheral nervous tissue and only later in the 1930s this phenomenon was termed with “perineuronal satellitosis” [1,2]. Further contributions came later by Brownson [1], Critchley, Andrew and Brain [3], Riese [4], showing such a numerical increase in satellite cells in normal aged neurological specimens as well as in young normal brains [1,5]. This increase, which does not necessarily indicate metabolic failure or even necrobiosis of neurons, suggests a vitalizing-like event, a perfect example of the symbiotic relationship standing between neurons and glial cells [2]. Most of the time, it indicates a condition that characterizes aged but essentially normal brain specimens. The *perineuronal satellitosis* phenomenon has also been described in healthy tissues of diverse brain regions such as cerebral cortex, hippocampus, basal ganglia and thalamus [1,2,6]. Although these aggregations of glial cells are frequently reported in physiological conditions, they are more commonly known as a histological marker of various pathological conditions within the central nervous system (CNS), for example, those associated with type I neurofibromatosis (NF1) [5], and they have been mostly recognised as histopathological markers of diffuse neoplasms such as various grades of astrocytoma and oligodendroglioma [6,7,8,9,10].

The first description of perineuronal and perivascular *satellitosis* in brain tumours was made by Hans Joachim Scherer, a pioneer in the study of glioma growth patterns, in his pivotal paper “Structural development in gliomas’’ [8]. After a careful revision of a certain number of glioblastomas (GB), he designated as *secondary structure* any form of tumour growth depending on a pre-existing tissue structure. He described thus seven types of secondary structures, plus an eighth type consisting of an eventual combination of two or more of the former ones: (I) perineural growth (also named neuronophagic growth), (II) surface growth, (III) perivascular growth, (IV) perifascicular growth, (V) intrafascicular growth, (VI) interfibrillar growth, (VII) white or grey matter growth. Each of these growth modalities, he observed, also had similar counterparts in specimens displaying findings of reactive rather than neoplastic processes and could potentially be related to glioma histological types and clinical prognosis. 

**Figure 1:**
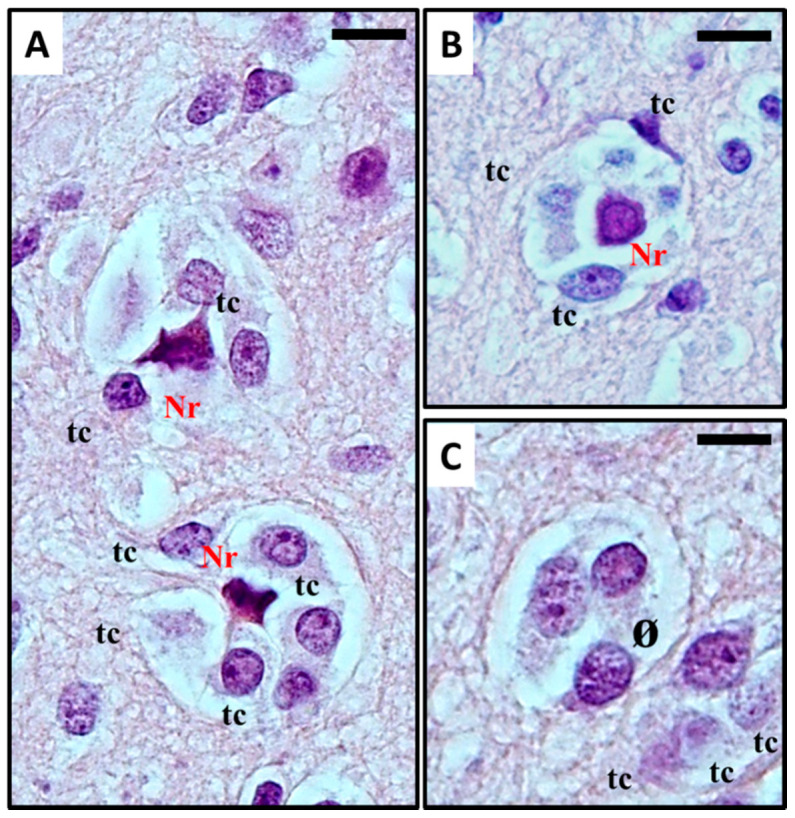
(**A–C**) Histological observation of *perineural satellitosis* in glioblastoma. Haematoxylin and eosin stain show (**A**) Anaplastic, frankly malignant glial tumour cells (tc) induced neuronal apoptosis (**B**): only the picnotic remnant (Nr) of the neuron remains. In the end, (**C**) the vanishing neuron will be completely replaced by tumour cells (ø). What normally occurs is that neoplastic cells surround the neuronal cell body, which then results in neuronal cell death with intracellular degenerative change and appearance of ‘ghost cells’ surrounded by the neoplastic cells in electron microscopy studies. The neoplastic cells can also phagocytose the remains of the neurons. For detailed information refer to [11,12]. Original images are collected at 400× magnification and relative region of interest (ROI) is reported. The scale bars are 20 µm.

Perineuronal growth, what we now define as *perineuronal satellitosis* (Figure 1A), is characterised by growth of neoplastic cells around a neuron’s cell body and dendrites. Sometimes, this growth leads to the replacement of neurons by groups of tumour cells (Figure 1B,C), a highly characteristic feature which Scherer himself termed “neuronophagic growth” [8,9,10]. 

**Figure 2:**
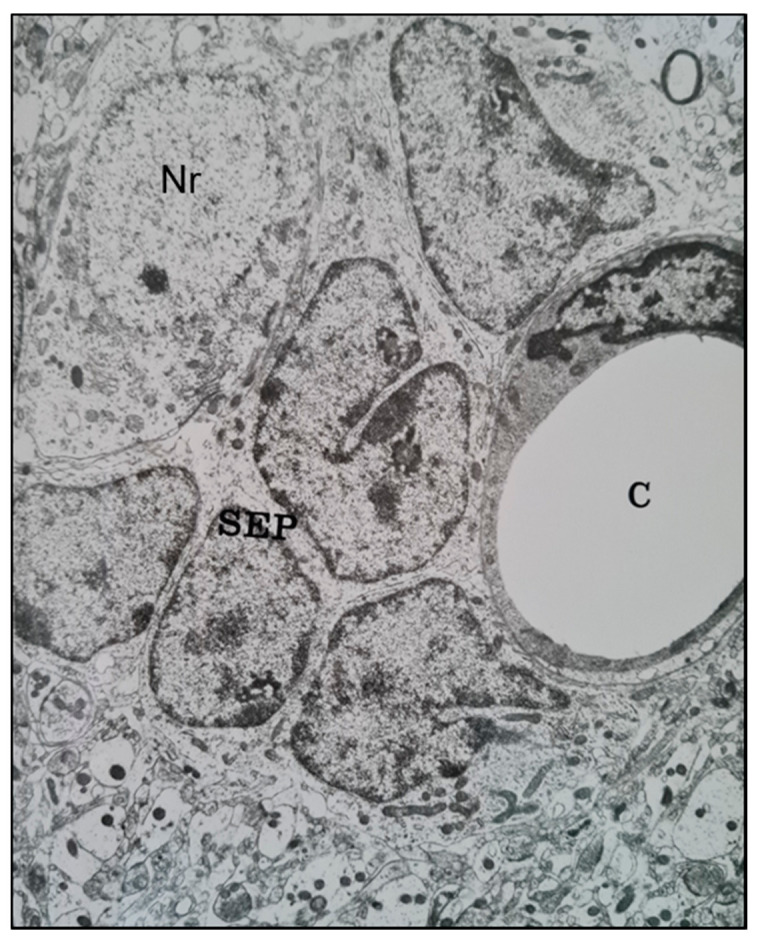
A transmission electron microscope micrograph of the sub-ventricular zone of the brain of a trans placentally ENU-treated BDIX rat showing neoplastically-transformed sub-ependymal plate glial stem cells (SEP) closely juxtaposed with a brain capillary (C) (right) and a neuronal cell body (Nr) (upper left). ×10,800.

Similar features were seen in Ethyl Nitrosourea (ENU)-induced gliomas in BD-IX rats where a range of neuronal degenerative changes whereby at the most severe manifestation “ghost cells” or dying neurons replaced normal neurons and these were surrounded by neoplastic glial cells [11,13]. Interestingly, both neuronal and perivascular *satellitosis* (Figure 2) have been demonstrated many years ago by sequential electron microscopic examination of the subventricular zone (SVZ, also known as the subependymal plate). In this rat model of glioma, pregnant rats were treated with a potent neuro-carcinogen, Ethyl Nitrosourea, on the 16th day of gestation and the resultant offspring developed glia neoplasms predominantly located adjacent to the lateral ventricles of the brain (a known ‘germinal’ zone). Thus the developmental genesis of such primary brain tumours could be followed and manifested both peri-neuronal and perivascular satellitosis as early hallmarks of developing brain tumours [7,12]. 

**Figure 3:**
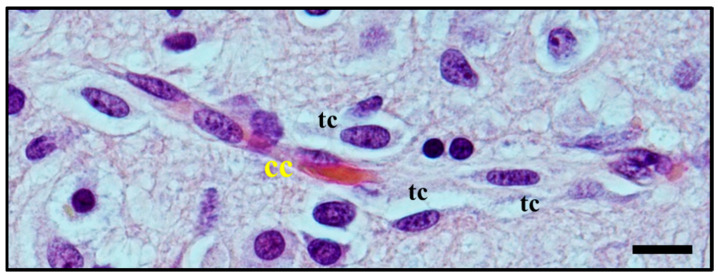
Histological observation of *perivascular satellitosis*. Atypical glial tumour cells (tc) moving and stretching along capillary vessels: increase in nuclear size of endothelial capillary cells (cc) is a sign of their metabolic reaction to tumour cells aggression and makes nuclei bulge into vessel lumen. Original image collected at 400× magnification and relative regions of interest (ROI) are reported. The scale bar is 50 µm.

Moreover, perivascular growth, what we now define as *perivascular satellitosis* (Figure 3) is characterized by the growth of neoplastic cells in Virchow–Robin spaces around blood vessels, typically small sized capillaries or precapillary vascular structures. As stated in an even earlier publication [8], Scherer observed how this form of growth was an early manifestation of tumour spread and probably one of the first pathways of neoplastic diffusion, yet tended to be present also in later stages, highly infiltrative areas of tumours, and usually was easier to detect in cortical and striatum grey matter than in white matter.

Given these preliminary studies on the invasive pattern of glioma, he postulated that a complete surgical excision of infiltrative glial neoplasms was technically impossible, thus explaining the high and fast recurrence rates not only of GB but also of lower grade glial tumours: this concept could be applied even at a time when hemispherectomies pioneered by Walter Dandy [14] were still being practised as standard treatment for glial neoplasms, and recurrences were observed in the contralateral hemisphere as early as six months postoperatively [15]. Despite these early speculations on the functional significance of *satellitosis*, the phenomenon has received very little attention in more recent years. In this review, we describe the biological crosstalk between glioma cells in *satellitosis* beyond the histopathological importance of these patterns into neurological diseases, particularly into the diagnosis and behaviour of primary brain tumours.

## 2. Satellitosis in Neurological Disease 

### 2.1. Histological Characterisation of Perineuronal Satellitosis in Brain Tumours

**Figure 4:**
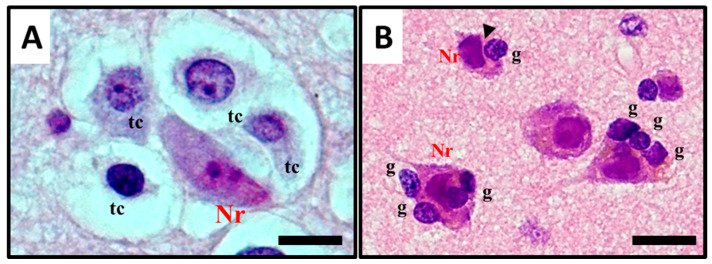
Histological observation and description of (**A**) perineuronal satellitosis in glioblastomas (GB) and (**B**) perineuronal satellitosis in non-neoplastic brain damage condition. (**A**). Polymorphic, markedly atypical glial tumour cells (tc) surrounding a neuron (Nr), which nevertheless shows only mild sign of cellular, mostly hypoxic stress (darkened colour of nucleus and cytoplasm, focal vacuolisation, chromatic dispersion); (**B**) Slightly atypical, non-neoplastic glial cells (g) are surrounding neurons (Nr) with little changes, mostly due to hypoxic stress, in the collateral brain tissue of an active plaque (patient with relapsing multiple sclerosis). The contact between the cell’s shapes, not only cytoplasmic but also nucleus structure, creating nuclear membrane indentations (►). All images are shown at 400× magnification and relative regions of interest (ROI) are reported. The scale bars are 50 µm.

The term *satellitosis* usually refers to an increase in the number of cells encircling a neuron. The term has been applied to both *reactive* and *neoplastic* processes. Sherer himself preferred the term *growth* to define the neoplastic ones in order to avoid confusion [8]. In everyday practice of neuropathology, *neoplastic satellitosis* (Figure 4A) is more commonly seen than *reactive satellitosis* (Figure 4B), typically in association with diffuse astrocytic neoplasms [15] and easily found when the tumour infiltrates the grey matter [16]. A common example of *reactive* process instead, can be exemplified by neuron degeneration, where the satellite cells are usually represented by microglial cells (Figure 4B) [17]. In order to recognise these patterns as microscopic features of neoplastic or reactive pathological conditions and to distinguish *perineuronal* malignant satellitosis from *reactive satellitosis* and microglial neuronophagia, the recognition of atypical nuclear morphology of infiltrating neoplastic astrocytes or even their glial nature could be difficult in the absence of immunohistochemical or ultrastructural analyses (Figure 1A). Also in other secondary structures described by Scherer, such as perivascular, subependymal, and subpial spread, satellitosis mirrors the ability for the infiltrating tumour cells to breach the glia limitans and enter the subarachnoid space: even at gross observation, typically during neurosurgeries for high grade glioma resections, neurosurgeons can easily recognize mounds of tumour on the surface of the brain, and clearly see the presence of a subjacent tumour [18].

Among all CNS tumours, gliomas represent a typical primitive entity, and, in particular, gliomas are frequently diffuse in nature [19]. Diffuse gliomas are distinctive due to their insidious pattern: according to the fourth edition of the WHO Classification of Tumours of the CNS [20], they represent a heterogeneous group of tumours characterised by a distinctive, infiltrative growth of surrounding neuropil and CNS structures. Based on both molecular and histopathological analysis, diffuse gliomas are subdivided in astrocytic and oligodendroglial tumours (the diagnosis of mixed oligo-astrocytic neoplasms being currently strongly discouraged by the WHO) and graded as low grade (WHO grade II) or high grade (WHO grade III and IV). Providing an accurate distinction between the different diffuse glioma types and malignancy grade has a significant impact on prognosis and therapeutic response [21]. Unfortunately, they all carry a fatal prognosis, even when treated with the most advanced protocols of chemo- and radiotherapy combined with surgery [22,23].

Diffuse gliomas often appear to arise as subcortical white matter masses, subsequently diffusely infiltrating the cortex and other grey matter areas. Diffusing lesions display variably atypical tumour cells, organising themselves into secondary structures (predominantly perineuronal satellitosis in more than 90% of cases) and causing the formation of regressive structures such as calcifications and/or microcysts in surrounding tissue [24]. Recognition of extensive, diffuse infiltrative growth in contiguous normal brain tissue structures is of great help in the diagnosis of diffuse glioma, while at the same time explaining the predictability of tumour relapse associated with this kind of neoplasm. Involvement of the cortical grey matter is a highly distinctive feature of diffuse tumours [25]. Within the grey matter, tumour cells are frequently found immediately adjacent to neuronal cell bodies, where they are often referred to as *satellite cells* (satellite cells within the sensory ganglia of the peripheral nervous system are present in large numbers but represent Schwann cells) (Figure 1A) [1,2,6,24,25,26,27]; although immunohistochemical stains for myelin-associated glycoprotein (MAG) and for myelin basic protein (MBP) have been used to reveal oligodendroglia cells, with varying success they did not reveal reliable or reproducible results. In this area instead, *perineuronal satellitosis*, perivascular aggregations of tumour cells (Figure 3), and subpial accumulations may be noted and provide valuable ancillary clues to the diagnosis [21,28]. Microscopic examination shows that the tumour cells tend to invade individually or in small groups in the neuropil, (the network of neuronal and glial cell processes in grey and white matter). Only a few neoplasms such as metastatic lymphomas and occasionally metastases from small cell lung carcinoma display such pattern of growth in the CNS [27]. Despite these early findings related to neoplastic glial cell invasion and neuronal satellitosis, the direct growth-promoting effects of active neurons in the tumour microenvironment and the mechanistic details in primary brain tumours and other cancers has not been fully described [28]. Indeed, the question whether neuronal activity can promote glioma progression and eventually how efficient this promotion may be has been investigated for many years without a true answer being found. Nowadays, an increasing body of evidence suggests a possible role of neurons and neuronal activity in fuelling glioma growth and neoplastic cell invasion [28,29,30,31,32,33].

### 2.2. Molecular Mechanisms Involved in Perineuronal Satellitosis Phenomena

Many studies investigated the role of non-neoplastic glial cells in GB growth and evolution [34,35,36,37,38], proposing different mechanisms [32,33], which may even contribute to drug resistance [33]. However, increasing evidence supports the hypothesis that precursor glial cells, glial cells and glioma cells may mutually benefit the close relationship with surrounding neurons [28]. Among glial cells, astrocytes play a pivotal role in CNS tumours [34,35] as well as in physiological events such as neurotransmission processes [36] water homeostasis, defense against oxidative/nitrosative stress, energy storage, mitochondria biogenesis, scar formation, tissue repair via angiogenesis and neurogenesis, synapse modulation by regulation of neurotransmitters and ions concentrations [37]. Astrocytic cells are actively involved in neurotransmitter recycling, clearance of extracellular potassium ions, and in the propagation of Ca^2+^ waves [38]. The additional role of astrocytes as a source of energetic fuel for neurons was originally proposed by Pellerin and co-workers (1994), who described an activation of neurons with subsequent release of the neurotransmitter glutamate stimulating glycolysis in nearby astrocytes: the lactate produced in this glycolytic burst is released back to neurons and pushes neuronal metabolism further. The hypothesis has been termed the “Astrocyte-Neuron Lactate Shuttle Hypothesis” (ANLSH): such a model does not exclude a direct neuronal glucose uptake. However, it suggests that lactate produced by astrocytic glycolysis is the main metabolic fuel of glutamatergic neurons during neurotransmission [39]. Although the hypothesis focuses on the mechanism by which astrocytes may “talk” with neurons, we could pave the way for the hypothesis that neoplastic astrocytes can interact with neurons in the same way. Pei et al. [40] reviewed the implication of neurotransmitter molecules on glioma progression, underlining how glutamatergic and calcium (Ca^+^) signaling exerts a positive feedback on glioma development by metabolic reprogramming, which accelerates glioma growth. 

Based on this earlier speculation Civita et al. [41] have analyzed the histological importance of *perineuronal satellitosis* in human GB tissues. The authors claim to provide the first evidence of single cell LCM RNA-seq of different regional compartments within “de novo” IDH1-wt GB samples, particularly glial cells cuffing neurons and the neurons themselves reporting how satellite cells show an up-regulation of genes related to integrins, specific metalloproteinases, aquaporins and cell division control protein 42 homolog (CDC42) signalling, which are known to be involved in invasion and glioma aggressiveness [42,43]. Notably, they report that the cells collected in *satellitosis* areas show an overexpression of BRCA1, SPARCL1, MMP9 and MMP28 genes associated with GB malignancy [43,44,45]. Moreover, the over-representation of metabolic pathways (i.e., the TCA cycle) in both compartments showed up not only an anchorage-dependent role of neuronal somata but a mutual exchange of metabolic signals between cellular elements later identified as neoplastic cells [41]. However, the limited number of samples, as well as the minimal quantity of tissue used, require further studies to confirm these findings. 

Zagzag D et al. in an elegant study [46] described how, under the stimulus of hypoxia and Vascular Endothelial Growth Factor (VEGF), stromal cell-derived factor 1 alpha (SDF-1α), also known as C-X-C motif chemokine 12 (CXCL12), expressed by neurons, blood vessels, subpial regions, and white matter interacts with its own receptor expressed by glioma cells, inducing tumour cell migration in a chemokine receptor type 4 (CXCR4) and 7 (CXCR7) anchorage-dependent manner [47]. This phenomenon was one of the first descriptions of how the secondary structures of Scherer have a molecular basis and should not be considered a casual feature of tumours. 

In different pathological conditions such as stroke and brain ischemia, neural energy depletion is accompanied by a massive release of glutamate [48]. It has been reported that glutamate and Alpha ketoglutarate (α-KG) are key elements and fuel of glioma metabolism [49,50]. In the brain TME, the glutamate released by neurons acts in a migration-promoting way (i.e., as an ‘enhancer’) [51,52] enhancing tumour cell spread within the brain parenchyma and implicated in brain tumour metastasis, particularly breast to brain metastatic growth [29]; the N-methyl-D-aspartate (NMDA) receptor signalling on breast metastatic cells may influence the uptake of glutamate from neurons and promote tumour cell proliferation, thus proposing a rationale for brain metastatic growth. All together these data support the idea that glutamate may have a key role in glioma progression as well: (I) by acting as an excitotoxin, it clears space for tumour spread thereby promoting tumour growth, (II) by acting as an “enhancer”, promoting cell motility and consequently promoting tumour invasion. 

It is also known that glioma cells release glutamate by themselves [51] acting locally by leading tumour growth [52,53], inducing excitotoxic activity [54], and causing cellular oedema [55]. High levels of glutamate have been implicated in numerous seizure disorders [56], including glioma [57,58,59,60]. Studies conducted in both glioma patients and animal models have suggested that epilepsy activity originating within the peritumoral edge, 1–2 mm away from the tumour mass, is related to invading tumour cells surrounding neurons [61,62,63], showing how the epileptiform activity is more pronounced in tumour-invaded neocortex. These phenomena explain why many patients’ seizures are an early clinical sign and over 80% of glioma patients suffer seizures during the course of the disease [60,64]. Several ongoing and completed clinical trials are exploring the rationale for pharmacological targeting of glutamate receptors and transporters to interrupt crosstalk in glutamate-mediated brain tumour growth.

More recent studies have also emphasised the role of synaptic input to brain tumour [65], in particular of neuronal activity which in the adult brain induces neuroglial stem and progenitor cell proliferation, and leads to the migration process via glutamatergic synapses [66], thus a similar process could be adopted by glioma cells. 

**Figure 5:**
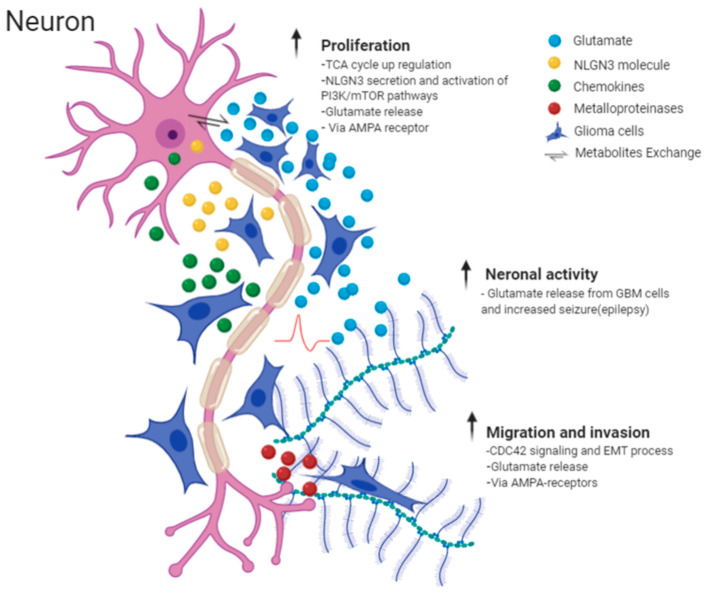
Illustrative picture of Perineuronal satellites events and major molecular mechanisms, which explain the interaction between neurons and neural activity and glioma cells. Images are created with BioRender.com.

On a similar theme, Venkatesh et al. [67] in one of his extensive murine studies explored the influence of neurons on glioma growth and demonstrated that certain cells, pyramidal neurons, promote proliferation via the PI3K/mTOR pathway in adult and paediatric high grade glioma (HGG) cell cultures by secreting neuroligin-3 (NLGN3) [67] and targeting NLGN3 could prove a basis for a promising therapy in HGG [68]. The same group later showed that peritumoral neurons and glioma cells directly interact through AMPA-receptors that drive tumour proliferation and invasion [69] by facilitating oncogenic signalling cascades and cytoskeletal remodelling. Major findings are summarised in Figure 5 and Table 1.

Many strategies have been tested using in vivo models (summary Table 1) in order to study the interactions between glioma cells and non-glioma brain cells, but lack of specific molecular markers to clearly distinguish these two cell types and directly characterise their interactions in vivo for short to long periods during glioma development have delayed knowledge on this interaction. Nevertheless, evidence provided about the ‘back-and-forth’ of normal and malignant neural circuitry could hold promise for targeted therapies to treat these devastating diseases. 

### 2.3. Histological Characterisation of Perivascular Satellitosis in Brain Tumours

Tumour cells have long been known to connect with vascular structures, both from the tissues they stem from and from infiltrated or metastasized ones. The first morphological evidence of this interaction was reported at least as far back as 400 BC and 192 AD, respectively, by Hippocrates and Galen [71]. It was John Hunter in 1787 that introduced the term *angiogenesis* and related it to inflammatory processes [72]. While the first report of blood vessels apparently stemming within tumours was reported by Rudolf Virchow in 1863 [73], when he showed that solid tumours have their own blood supply, a few years later Thiersch (1865) and subsequently Goldmann (1908) provided more precise descriptions of tumoral vessels as vascular neo-formations featuring high proliferation and both chaotic and irregular growth [74,75], a description that may fit with the concept of tumour angiogenesis. 

Early observations of perivascular patterns surrounding the Virchow–Robin spaces of pre-existing brain vessels were reported by Scherer [8,9]. In his studies he reported that 35% of gliomas, both in earlier and later disease stages, form cuffs of glial neoplastic cells surrounding capillaries and small vessels of the brain. Later, he went on to recognise this process of “perivascular gliosis” as one of the distinctive features of glial tumours [9]. Moreover, he pointed out how the organisation of glioma cells tends to turn into the formation of cell cuffs around normal micro-vessels typically found in areas of apparently normal brain parenchymal tissue at some distance from the original tumour mass, and he underlined the precocity of the process in the biological history of the neoplasm [8]. This early finding about *perivascular satellitosis* has inspired many groups over the years, which described the phenomenon using a wide range of different terminologies, particularly “vessel co-option”. Even though researchers use this term to describe the molecular mechanism where glioma cells reach and subsequently encircle vessels, the term *perivascular satellitosis* is still used by pathologists and is currently reported in textbooks to teach the main histological features of vasculature in pathological conditions.

*Perivascular satellitosis* has been reviewed in many tumours by Kuczynski et al. [76] highlighting the key histopathological traits associated with this process in cancer, while also clarifying the terminology used for different processes. 

**Figure 6:**
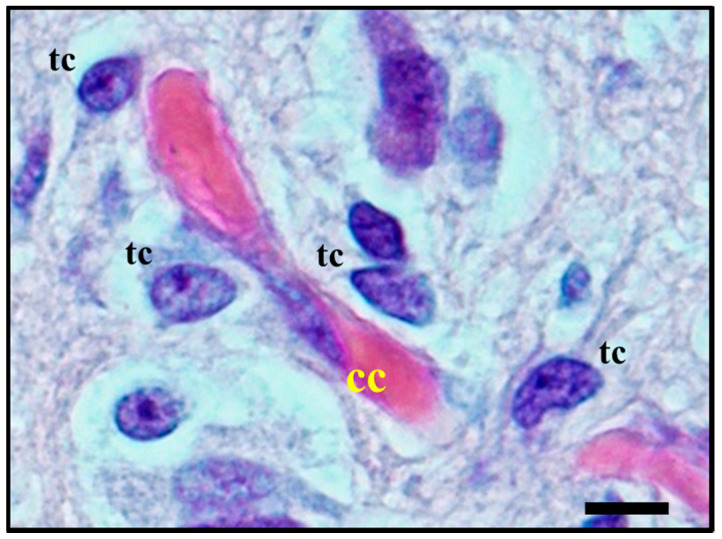
Histological observation of *perivascular satellitosis* shows atypical glial tumour cells (**tc**) moving and stretching along capillary vessels: a marked increase in nuclear size of endothelial cells (**cc**) is a sign of metabolic reaction to tumour cells aggression. The original image is captured at 400× magnification with a relative region of interest (ROI) reported. The scale bars are 50 µm.

In glioma, the term *perivascular satellitosis* is used to refer to a non-vasculogenic process whereby blood vessels hijack glioma cells to migrate towards pre-existing vasculature (Figure 3 and Figure 6) [77]. This feature has commonly been found in both high and low-grade glioma [15]. In high-grade glioma cells entirely surround the blood brain barrier (BBB) capillaries (perivascular growth pattern), modulating the function of pericytes and the properties of the BBB, while, in low grade glioma, vessels are surrounded when the cancer cells infiltrate the brain parenchyma (diffuse infiltrating pattern) as single cells in proximity to vessels.

### 2.4. Molecular Mechanisms Involved in Perivascular Satellitosis Phenomenon

The mechanisms that trigger neoplastic “satellite” glial cells to migrate and invade are multiple and interdependent [78]. 

Recent works [79,80] adopting new advanced intravital microscopy imaging technologies have recorded patient-derived GB cell movement in mouse brain, showing by time-lapse how GB cells closely interact with the microvasculature of BBB and they move along the pre-existing vasculature of the brain. Here, neoplastic cells can travel several millimetres or even centimetres away from the main tumour mass. Interestingly, during this invasion process the infiltrative cells, so-called guerilla cells, protected from cytotoxic agents by an intact BBB, invade normal brain; in particular, it has been seen that neoplastic cells adhere to vascular basal laminae, where mitogenic growth factors are sequestered within the extracellular matrix components stimulating glioma cells to divide, suggesting that this process that takes place around capillaries is a possible form of ‘pseudoinvasion’ [78]. 

A recent review by Seano et al. [81] discussed this phenomenon proposing two mechanisms: (I) individual-cell co-option; and (II) collective-cell: vessel co-option mechanism [82]. The same group also demonstrated the process behind the collective-vessel co-option that causes disruption of the astrocyte: vascular coupling and blood-brain barrier (BBB) breach, with consequent blood vessel leakage, abnormal vasculature (large lumen and tortuous architecture) and later inflammation. While individual cell co-option is led by astrocyte-like GB cells (i.e., Olig2- and Wnt7-negative) via Olig2-Wnt7, a signalling axis [80] does not involve an inflammatory process and spread in association with blood vessels; this later process is undetectable even with advanced imaging techniques [83]. Moreover, the authors raised the possibility that these mechanisms, although still unclear, might be related to the failure of current anti-angiogenic treatments. 

A number of mechanisms driven by diverse molecules have been proposed (summary Table 2) to explain *vascular satellitosis* in mouse models. A few of these models were based on patient derived GB cells [77,80,84,85,86] through in vitro cell culture, due to lack of relevant alternative models as well as the high complexity of the GB microenvironment, particularly its marked tissue heterogeneity [87]. 

Previous studies from Holash [97] and Gale [88] aimed to understand the mechanism based on the enrolment of cancer cells along pre-existing vessels, showing how tumour cell migration towards perivascular sites is related to the expression of pro- and anti-angiogenic endothelial growth factors, for example, angiopoietin-1 and 2 (ANGPT-1, ANGPT-2) and VEGF. Interestingly, they also observed this process using rat mammary adenocarcinoma and then by injection of lung carcinoma cells intravenously, which reach the brain parenchyma, cuffing normal brain blood vessels. This study was later supported by Kusters [89] with further experiments using melanoma cells. Notably, he observed a colonisation of melanoma cells within healthy brain parenchyma and the VEGF-engineered melanoma cells hijacked pre-existing vessels without inducing any angiogenic process or sprouting of new vessels. 

The deep interaction between glioma and vascular structures assures a continuous supply of oxygen and nutrients essential for cell growth while glioma stem cells are exposed to a variety of growth factors, chemokines, cytokines, and kinins. 

Considering the role of the microenvironment on neural stem cells fate [98], Calabrese et al. [99] have shown for the first time the presence of a vascular niche that regulates brain tumour stem cells. This GSC niche is characterized by endothelial cells that interact closely with brain tumour cells maintaining these cells in a stem-like state. Using mouse orthotopic model, they have observed that increasing the number of endothelial cells or blood vessels increases the fraction of self-renewing cancer cells around the vascular structures, thus demonstrating the direct involvement of the vascular structure into creating a favorable microenvironment for self-renewing of GSCs and tumour growth. Although pioneering in the field of GCSs, the work from Calabrese et al. did not specify the type of vasculature involved in these processes. 

Later Hira et al. [91] claimed to demonstrate that the perivascular niches of GSCs involve arterioles, not capillaries. By immunohistochemistry, they confirmed that CD133-positive and nestin-positive GSC cells reside in hypoxic environments surrounding CD31-positive endothelial cells (ECs) and smooth muscle actin (SMA)-positive smooth muscle cells of arterioles. Moreover, those GSCs express not only a SDF-1/CXCR4 axis but also osteopontin (OPN) and cathepsin K (CTSK), showing how the niche that surrounds arterioles resembles bone marrow hematopoietic stem cell (HSC) niche proteins and recruits glioma stem cells by promotion of migration via CD44 and CXCR4 [91,92,100]. 

In pursuit of these themes, Zagzag D and his team [46,47] have shown, in different works, how the up-regulation of (SDF)-1α and CXCR4-receptor as well as CXCR7 on glioma cells promotes cell migration towards blood vessels through a saltatory process: this process includes periods of immobility, during which glioma cell divisions take place near vascular branch points, suggesting that the mitotic process and invasion are also triggered by local environmental cues. 

Montana et al. [90] have shown how signals such as bradykinin promote the chemotactic attraction and invasion of glioma cells that express bradykinin 2 receptors (B2R), proposing a B2R antagonist as a future anti-invasive drug approach in glioma therapy.

More recent studies have also emphasized the role of GSCs in colonization of the perivascular structure. In two distinct studies, co-culture of patient-derived GB and endothelial cells was used to discover the chemotactic pathways activated by endothelial cells to stimulate GB cell invasion. The authors showed that endothelial interleukin-8 (IL-8) increased GSCs invasiveness and growth [94,101]. A clear example of crosstalk between glioma cells and components of BBB was also reported by Caspani et al. [83], which showed that close interaction between tumour cells and pericytes gives rise to GB cell/pericyte fusion-hybrids in a CDC42-dependent manner and promotes tumour diapedesis. Cell-to-cell communication between glioma cells and perivascular cells has been documented by use of intravital imaging in a murine model of GB [85]. The authors showed how the endothelial cells, via overexpression of Ephrin-B2, lead glioma cells to surround vessels, thus the downregulation of this process may improve patient survival. 

**Figure 7:**
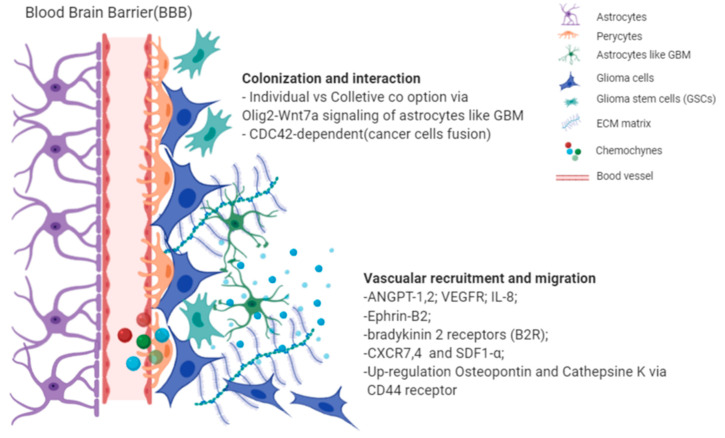
Illustrative picture of *perivascular satellites* events and major molecular mechanisms, which explain the interaction between endothelial cells from BBB and glioma cells. Images are created with BioRender.com.

At least five different neovascularisation processes have been identified in GB: (i) vascular co-option, (ii) angiogenesis, (iii) vasculogenesis, (iv) vascular mimicry, and (v) glioblastoma endothelial cell trans-differentiation [102]; the underlying biological mechanisms and classification of human tumours based on these particular processes are still unclear. All major findings and molecular mechanisms are summarized in Figure 7 and Table 2.

Diffuse invasion of the brain parenchyma along pre-existing blood vessels, could be a leading cause of therapeutic resistance, but the mechanisms—in particular those of cell adhesion to extracellular matrix proteins and mitogen-stimulated neoplastic cell division—remain unclear in GB, while in other cancers this knowledge has revolutionized prognosis [103].

## 3. Concluding Remarks 

Up to date, satellitosis has been variously discussed as an independent entity in brain tumours: this misconception leads to underestimating the significance of this histological feature, and prevents us from completely understanding its importance in disease progression and its potentially crucial role in the discovery of novel therapeutic strategies. The findings shown above report the current knowledge regarding the particular histo-morphological entities of perineuronal satellitosis and perivascular satellitosis: neither of them are randomly occurring formations, but indeed demonstrate that glioma cells have specific forms of tropism for particular brain structures. Unfortunately, our understanding of them is hampered by the limits of our experimental models, since neither patient-derived cell lines nor rodent cell lines can currently give us a fully reliable model of how tumour cell invasiveness towards any surrounding/stromal tissue happens and what it finally means [104]: this problem remains a huge obstacle, creating significant difficulties in the development of effective therapies against such aggressive neoplasms as diffuse gliomas. 

Recent developments in advanced MR and PET scanning have improved CNS imaging, up to the point that now we can collect specific information within brain TME compartments [105,106,107]. Nevertheless, a careful histo-morphological evaluation of tumour tissue by pathologists is still of fundamental importance, and features such as Scherer’s secondary structures are still a valuable clue into the diagnosis of diffuse, possibly high-grade gliomas. 

New, highly sophisticated in vitro models, stemming from either patient-derived tumour cells or pluripotent stem cells, could provide a scalable in vitro model to study diffuse gliomas [108,109] or metastatic brain cancer [110], although both with such limitations due to the lack of stromal component including vasculature. An effective matching of these models with a detailed pathological analysis, based on the combination of histomorphology and molecular parameters, should be employed to scan through glial neoplasms genetics and metabolic heterogeneity, in order to better understand TME complexity and eventually assess the predicted efficacy of targeted therapies [111]. 

## Figures and Tables

**Table 1:** Summary of studies investigating the *Perineuronal Satellitosis* process within brain tumour in in vivo and in vitro models along with the molecular mechanism proposed. Bold: is a summary title which encompass different process.

Experimental Models	Molecular Mechanism Proposed	References
Human Tissue sample and Animals	**(First evidence) Histological visualisation** - “Neurophagic growth” substitution of tumour cell;	[1,2,3,4,5,6,8,9,11,13]
Human Tissue sample	**Metabolic exchange and migration** - Upregulation of TCA cycle and transmembrane transport of small molecules; - CDC42 signalling and up-regulation BRCA1, SPARCL1, MMP9, MMP28 and aquaporin (AQP1 and AQP4) as well as EMT processes	[41]
Animals (in vitro, in vivo)	**Chemotactic attraction and migration** - VEGF, SDF1alpha, CXCR7 anchorage-dependent manner;	[46,47]
Animals In vivo, in vitro Patient derived GB cells	**Metabolic exchange and spared within brain parenchyma** - Glutamate released by neurons acts: (I) “enhancer”; (II) excitotoxin; - NMDA receptor on cancer cells uptake glutamate from neurons;	[29,51,52,53]
Human In vivo; in vitro Patient derived GB cells	**Neuronal activity on glioma promotion** - Increase neuronal activity by Glutamatergic synaptic; - Secretion of NLGN3 induce GB proliferation via PI3K/mTOR; - AMPA-receptors drive tumour proliferation;	[66,68,69,70]

**Table 2:** Summary of studies investigating the *Perivascular Satellitosis* process within brain tumour in in vivo and in vitro models along with the molecular mechanism proposed. Bold: is a summary title which encompass different process.

Experimental Models	Molecular Mechanism Proposed	References
Human, Animals in vivo	**First histological description** Cuffs of glial neoplastic cells surrounding pre-existing capillaries	[8,12]
Animals (in vitro cells, in vivo)	**GB cells interact with BBB:** Olig2^+^ oligodendrocyte precursor-like glioma cells invade microvasculature by single-cell via Wnt*7a/7b*	[80]
Animals (in vitro cells, in vivo)	**Mechanism of Individual co-option and Collective co-option:** Astrocyte-like GB cells interact with microvasculature via Olig2-Wnt7a signalling axis	[80,81]
Animals (In vivo cell lines)	**Vascular recruitment** - Pro Angiogenic VEGF and anti-Angiogenetic ANGPT1 balance	[88,89]
Human /Animal (In vivo; in vitro Patient-derived-GB, Tissue sample)	**Chemotactic attraction and invasion** - Upregulation SDF-1α and CXCR4-receptor as well as CXCR7 in GSCs with increase of OPT and CTSK; - Bradykinin-bradykinin 2 receptors (B2R) interaction; - endothelial IL-8 increased GSCs invasiveness and growth - invasion and cooption depend on IRE1α endoribonuclease activity	[90,91,92,93,94,95,96]
Human/Animals In vivo; in vitro	**GB cell/pericyte fusion-hybrids** Pericyte fusion-hybrids CDC42-dependent manner promoting tumour diapedesis	[86]
Human In vivo cellls; in vitro	**Surrounding microvasculature and migration** Upregulation of Ephrin-B2 lead glioma cell migration towards vessels	[85]
